# Using *k*-NN to analyse images of diverse germination phenotypes and detect single seed germination in *Miscanthus sinensis*

**DOI:** 10.1186/s13007-018-0272-0

**Published:** 2018-01-17

**Authors:** Danny Awty-Carroll, John Clifton-Brown, Paul Robson

**Affiliations:** Institute of Biological, Environmental and Rural Sciences, Aberystwyth University, Gogerddan, Aberystwyth, SY23 3EB UK

**Keywords:** *k*-NN, *Miscanthus*, Seed, Machine learning, Classification, Germination, Image analysis, Robust classification, Bio-energy, Seed imaging

## Abstract

**Background:**

*Miscanthus* is a leading second generation bio-energy crop. It is mostly rhizome propagated; however, the increasing use of seed is resulting in a greater need to investigate germination. *Miscanthus* seed are small, germination is often poor and carried out without sterilisation; therefore, automated methods applied to germination detection must be able to cope with, for example, thresholding of small objects, low germination frequency and the presence or absence of mould.

**Results:**

Machine learning using *k*-NN improved the scoring of different phenotypes encountered in *Miscanthus* seed. The *k*-NN-based algorithm was effective in scoring the germination of seed images when compared with human scores of the same images. The trueness of the *k*-NN result was 0.69–0.7, as measured using the area under a ROC curve. When the *k*-NN classifier was tested on an optimised image subset of seed an area under the ROC curve of 0.89 was achieved. The method compared favourably to an established technique.

**Conclusions:**

With non-ideal seed images that included mould and broken seed the *k*-NN classifier was less consistent with human assessments. The most accurate assessment of germination with which to train classifiers is difficult to determine but the *k*-NN classifier provided an impartial consistent measurement of this important trait. It was more reproducible than the existing human scoring methods and was demonstrated to give a high degree of trueness to the human score.

## Background

The use of image analysis techniques has been increasing in the biological sciences, offering high-throughput, unbiased and quantitative measurements [1] with reduced errors [2], but at the expense of real time interaction with samples. The slower set up but faster observations make image analysis ideal for time course studies [3], such as growth or germination, and the use of optical data makes such analysis ideal for calculating visual attributes such as plant size non-destructively, as in the case of field or automated glasshouse biomass assessments. This phenotyping technology lags behind that of genotyping technologies; however, it is increasingly being implemented to test or screen highly varied genotypes [4].

*Miscanthus* is a leading bio-energy crop and has a number of highly favourable attributes including a high net energy balance and the ability to grow on marginal land. It is not a food crop and therefore does not compete with food production unlike other potential bioenergy crops such as maize and Sugar Beet [5–7]. Most *Miscanthus* is grown from pieces of *Miscanthus* $\times$ *giganteus* rhizome which is a slow and expensive method of propagation especially at high numbers; therefore, to expand *Miscanthus* production seed based *Miscanthus* hybrids are being developed [8]. Seed-based propagation has the potential to rapidly increase the propagation rates and reduce planting costs [9]. *Miscanthus* seed are small, heterogeneous due to outbreeding [10, 11], with low germination rates at low temperatures [12] and therefore to improve seed propagation our understanding of seed biology and the control of germination in particular in this species must be improved.

Germination of seed is frequently scored by eye when the radical has visibly emerged [13, 14], this should allow embryo protrusion to be consistently scored by different researchers [15]. However, when using small seed and high numbers of samples, counts are less repeatable and less true. A computer system that is able to impartially score germination in a repeatable and reproducible way, would remove unknown variation from human-based scoring. A computer vision system perfects repeatability, possibly at the expense of trueness, which is an acceptable compromise in biological studies in which the relative impact of different factors on germination is important. Using photographs or other automatically recorded data for analysis, the algorithm can be refined and re-run on the samples in the future potentially by multiple research groups. Recording all the data digitally makes the collection of data faster and more reliable , particularly as a human scorer can be affected by time of day, repetition, and tiredness.

Automated systems such as MARVIN (GTA Sensorik GmbH) are often used for the accurate sizing and counting of seeds [16–19]. Measuring germination is more challenging; because depending on the experimental treatment, seed may not be sterile leading to mould growth, which may confound image analysis of radicle growth in scoring germination. Seed should be imaged repeatedly in the same position allowing algorithms to identify minor changes, and to disregard changes associated with mould or seed expansion due to water uptake, which should not be scored as germination.

Computer imaging of seed germination has been used to assess germination in *Arabidopsis* in comparison with human assessments [20]. A threshold (a set value used to screen out pixels) was applied to images to remove the background, the remaining objects were analysed in a selected colour range (e.g. RGB) and information about the seed’s average shade and perimeter determined. Parameters describing each object were collected and analysed simply and a distinction made between seed coat and whole seed including a radicle if present. Such methods have the potential to assess germination faster and with greater reproducibility than a human observer [20] provided the method uses only a final seed image and no initial photograph is needed for comparison. Using the difference between the object at different thresholds, germination can be scored with a high trueness to a human reference point [20]. The drawback to single image analysis is that the thresholding process needs to be very precise to achieve two images from one photograph that only differentiate the features such as the radicle or hypocotyl that are indicative of germination [20].

By using the idea of a ground truth, Ducournau et al. [21] was able to use receiver operating characteristic (ROC) curves to highlight the best strategy for producing data true to human vision; however, a significant unknown is the inaccuracy or bias of the human germination scores with which image analysis is compared. The ability to score different seed types depends upon experience and may be affected by mood and time constraints [22]. To compare the computer’s ability directly against that of a human may be unfair because the human is not necessarily an indicator of the real value; yet currently there is no more accurate method of determining the real germination score. Ducournau et al. [21] used mean time to 50% germination as the primary factor of comparison between the computer and the human analysis. In doing this, a seed-by-seed comparison of germination scoring between people and computers was avoided to create a fairer comparison.

In this study we combine the use of computer image analysis, ROC curves and machine learning to assess phenotypically diverse seed germination in comparison with a large set of human assessed images. A *k*-nearest neighbour (*k*-NN) method [23] was chosen as an efficient machine learning method [24] that could be implemented in R with the ‘class’ package [25]. *k*-NN works by finding each point’s nearest neighbours in an *n*-dimensional Euclidian space, then grouping that point with the *k* neighbours with which it is most closely associated [24, 26]. Tree-based algorithms were also considered but discounted because *k*-NN works with two categories and only two categories were needed (un-germinated and germinated) [27].

## Methods

**Fig. 1:**
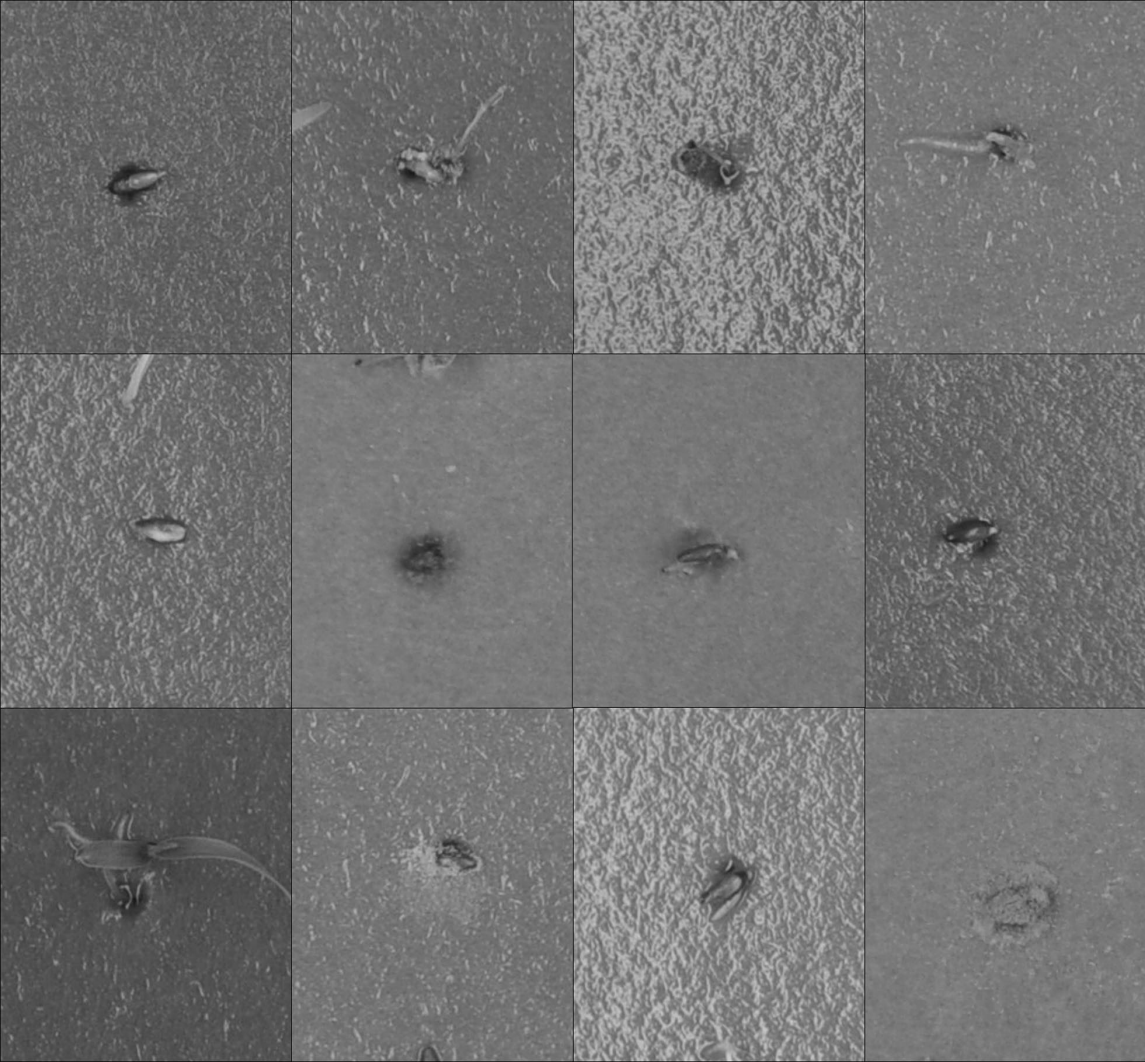
Example images of seed germination from the dataset. An example of twelve of the 16,896 seed images. These also show some of the problems for automation of germination scoring

A set of approximately 5000 *Miscanthus sinensis* seed germinating over 11 days, were photographed using a DSLR (Nikon D90) at a resolution of 282 $\times$ 341 pixels per seed image from an image of 4288 $\times$ 2848 pixels (see Fig. 1 for example of image data). The seed were sterilised with a low concentration bleach solution (0.5% Sodium Hypochlorite). They were then treated with standard plant hormones [gibberellic acid (from 0.15 to 750 mg $\hbox {l}^{-1}$), 1-naphthaleneacetic acid (from 0.01 to 200 mg $\hbox {l}^{-1}$), epibrassinolide (from 0.001 to 2 mg $\hbox {l}^{-1}$) and abscisic acid (from 0.05 to 60 mg $\hbox {l}^{-1}$)], or had induced water stresses (NaCl and polyethylene glycol (8000 and 4000 ppm respectively) producing water potentials of up to − 4.1 MPa) or they were stratified [28, 29]. Treatments were given no further consideration in this study because they were purely to produce a diverse and challenging range of germination phenotypes with which to test the image analysis. All images were scored by one person for consistency and the human score of this image set was the only reference point to which the computer score was compared. The images were analysed with FIJI [22], a distribution of ImageJ [30] customised for biological image analysis. Being common and open-source it has more flexibility to be used and developed by others than similar commercial systems. The images were processed through FIJI’s 3D object counter to identify size, position, and grey scale data (e.g. mean grey value) and the results for the central most object in each frame was recorded for analysis (image source [31]). The number of pixels at each RGB and HSB level was extracted in FIJI as histogram values for each image, and recorded with the other data.

A machine learning approach was used, as the non-ideal set of seed images used had been difficult to parameterise manually for image based germination scoring. The training data was loaded into an *n*-dimensional matrix, with *n* being the number of parameters e.g. size of seed object, object shade. The uncategorised data was added, and the parameters of each added datum were compared to all parameters in the training data. The *k* closest parameters by Euclidean distance (the nearest neighbours) were used to classify the new entry by majority vote. If an odd number is selected for *k* the vote will unambiguous, otherwise the tie is broken at random. Larger numbers of *k* produce more smoothing in the classification boundary [26].

This method was trained on a random set of half of the seeds and tested on the other half. This step was repeated multiple times to test and improve trueness by refining the value of *k* and the number of classifiers included in the training set. Traits from FIJI object detection (area, shade, etc.) were used as well as RGB and HSB histogram values for each thresholded seed object (e.g. R0–R255), to give a colour distribution for each image [32]. Because the absolute values of traits were across a several fold range, all traits were normalised to between zero and one. Due to the large number of traits, the image analysis was also tested after simplification to 21 component traits through a principle components analysis (PCA) (stats package: R [33]), this combined and summarised the main components of variation between images. An optimised subset of clear images (with no mould and only seeds that were distinctly germinated or not) that had been visually scored was also selected for use in the testing procedure. Each of these data sets—trait, trait with histogram, PCA, and idealised—were run *n* times to produce an average with a set of random splits of the data with an approximate 1:1 ratio of training to test data. All tests were run on a Intel® i7 2.8 GHz laptop with 64-bit Windows™ 7. Results were assessed using ROC curves, once these were calculated a combined score was determined to assess the final success of the *k*-NN methods once optimised. The final success of each method tested was determined using a single measure from the ROC, the area under curve (AUC), that was statistically equal to the chance the algorithm would rank a random germinated image more highly than a random un-germinated image [34].

The human scoring of time sequences produced what was expected to be an ideal score against which to compare. Pictures of seed from time zero (before the test started) were excluded from the *k*-NN method because this added an extra $\sim$ 5000 un-germinated images and their purpose as a starting point in the FIJI classification was not necessary for *k*-NN.

Due to the scoring of time sequences, once a seed was marked as germinated all images after that time in the sequence were marked as germinated. This resulted in a problem; seed images from later time points of seed that germinated and then died, and were originally scored by a human as germinated, would not appear germinated in isolation. To circumvent the problem the index of training data was reviewed by running the *k*-NN classifier and outputting the certainties (between 0.5—uncertain, 1—certain). The number of possible values was dependent on the value of *k*, so if all *k* of the nearest neighbours were the same the certainty would be 1 and if 4 of, for example, 7 nearest neighbours agreed the certainty would be 0.57. The images that were classified as least certain in each run were manually checked, and updated if necessary. Hereafter this set of image-identified germination amended by a human operator will be referred to as the ‘amended human assessment’.

The *k*-NN method was compared with ‘Germinator’, a standard package to automate germination detection devised by Joosen et al. [20]. 270 dish images (of 64 seeds per dish) were split into two groups for training and validation. The ‘Germinator’ method first optimises the scoring of un-germinated seed in the training data, before predicting the germination in the validation data. The use of individual seed images, as employed in the *k*-NN method, allowed for the calculation of the AUC from a ROC curve. This could not be achieved using ‘Germinator’ and thus exact comparisons of the methodology employed by the two methods could not be made; however, broad comparisons of speed and accuracy were possible.

## Results

For the main testing of the *k*-NN method, 16,896 seed images were used for which 25 variables from FIJI object detection (area, size of bounding box, mean median & standard deviation in shade, distance to centre of the object, width & height, etc.) and an additional 1536 variables from RGB and HSB histograms of the thresholded images were produced.

**Fig. 2:**
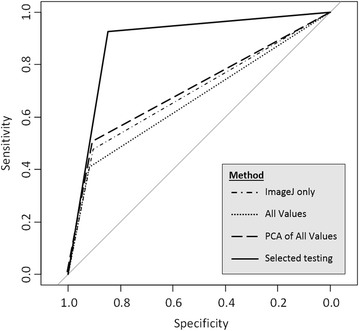
ROC curves using different methods. ROC curves from four tests of *k*-NN using different methods. The ImageJ only line uses only the 25 outputs of the ImageJ object detection (dash-dot). All values expands the data to all 1561 variables (to include the histogram values for RGB and HSB) for the classifier (dot-dot). The PCA of all values uses a PCA to reduce the dimensionality of the data to 21 principle components (dash-dash). An optimised image set used just the images that clearly demonstrated to a human un-germinated or germinated seed with the same 21 principle components (sold line). All results were generated using a random seed of 1234, to show one representative result

The *k*-NN classifier was tested using the 25 variables produced by FIJI’s object detection using the same 16,896 seed images. When assessed in comparison to the amended human assessment with a *k* value of 7 this gave an AUC for the ROC curve of 0.69, with 558/8394 (0.066) false positives and 1345/8394 (0.16) false negatives (Fig. 2). The runtime was 2.3 s. Histogram data was collected on each image and was used to add more data for the classifier. Using the resulting full set of 1561 variables (and thus producing a 1561 dimensional space to assess the seed) was computationally intensive for extensive testing (runtime of 3011 s); but for comparison one run with a *k* of seven resulted in an AUC for the ROC curve of 0.664 and 458/8394 (0.054) false positives and 1526/8394 (0.153) false negatives (Fig. 2).

The number of variables was reduced by PCA to the first 21 principle components which explained 70.8% of the variation. Because the PCA had reduced the number of variables for *k*-NN, the process could be run repeatedly, with a runtime of 183 s to produce the PCA and then 1.8 s to run the *k*-NN. This *k*-NN process was used to amend the human assessment where necessary until there were no more seeds for which an amendment was necessary. The *k*-NN was run against the amended human assessment (Fig. 2) and gave an AUC of 0.706 and 561/8502 (0.066) false positives and 1298/8502 (0.153) false negatives.

An optimised image set of 711 seed was tested and a subset chosen unevenly using a ratio of 1:2 to provide 233 test seed. This simplified the inputs to the 25 FIJI variables based on object detection. The *k*-NN gave a false positive of 8/233 (0.034) and a false negative of 19/233 (0.082) and an area under the ROC curve of 0.887 (Fig. 2).

In comparison analysing the images using ‘Germinator’ [20] took 3 h to train on a set of 141 images containing 9024 seeds, and 5 min to run on a validation set of 130 images with 8320 seeds. The training optimised to a cumulative difference in the total number of un-germinated seed of 1692 seeds out of 6728 human scored un-germinated seeds (25.1% different). In the validation set of images the total number of un-germinated seeds was 7.3% different from the total of the manual counts (412/5644), for the germinated seed this was 31.3% different (830/2656). In the 130 dishes of seed counted the number germinated was only the same as the manual count 5.4% of the time and on average the germination count for each plate was 10.5 seeds different than the manual counts.

## Discussion

This study of automated germination scoring through seed-by-seed analysis was tested on individual seeds using ROC curves, rather than score the number of seed germinated over the whole plate. Other studies have fitted curves to germination scores over a time series to compare the models of human counts to the computer assessed counts [20], or have tested scores against total emergence to determine if the system could arrive at the same conclusions as found using human scoring as an absolute standard [35]. In this study, the classification of individual seed is used as the measure of success rather than the model of a germination curve for a seed batch. By doing so this method tests the per seed accuracy of automated scoring.

While an exact comparison with an existing germination detection tool (‘Germinator’ [20]), which works on a “by tray of seeds” basis, was impossible, a comparison test using the original images of the whole seed trays was produced. The ‘Germinator’ method had a greater total run time than did the most complex of the *k*-NN tests, but speed was comparable once trained. The accuracy of this method was much less, and while the total number of un-germinated seeds were very similar (7% different), the total germinated count was less close to the human score (31% different). However, these values allow under and over estimation between dishes to balance out the result; estimations of the per seed error were much higher, being on average 10.5 seeds different from a manual count. The difficulty in the ‘Germinator’ assessment was possibly due to over prediction of germination from the early presence of mould, followed by under prediction due to small changes in early germination, then at later time points, poor scoring from inaccurately determining the number of seed on the dish, due to the presence of mould obscuring seed.

The most important factors in the application of computer vision for seed germination counting are reproducibility and speed compared with a human. If computer vision offers no advantage, there is no reason to switch from a manual assessment. All methods of pre-processing the data before using *k*-NN provided a trueness to the human score of at least 0.66 area under a ROC curve. With a large set of $\sim$ 16,000 seed images the method showed a robustness to other factors such as mould growth and changes in the size and colour of the seed over time. The human score cannot be defined as an absolute measure because it lacks reproducibility. The *k*-NN score is trained on the human score and is therefore also not an absolute measure but it does offer an impartial, reproducible and consistent measure. However, the *k*-NN method requires a large set of human assessed data for training, which is time consuming.

Germination is a function of time and a machine learning approach could utilise the time at which the picture was taken, which may make analysis more effective; however, this was not utilised in this study, because it would be difficult to weight the times correctly to avoid bias in the result. For example, if a seed lot had reached 80% germination by day six, the *k*-NN would have an 80% chance of being correct when reporting on any seed over day five. Essentially this could lead to a polarised distribution of false positives and false negatives, as early germinating seed would be more likely to produce a false negative, and un-germinated seed would be more likely to produce false positives at later time points. This would undermine the point of using machine learning on germination testing.

To assess the *k*-NN method, the human assessment of germination required adjustment. This was due to how the human assessment was produced, and demonstrates the shortcomings of human scoring. The best outcome achieved with the human scorers was on a sub sample of the seed for which germination state was clear to a human scorer. With this subsample of seed images, the *k*-NN achieved 0.89 (area under the ROC curve). In [36] the median time for 25 seeds to germinate had a standard deviation of 0.8 h on average between human scorers over 18 dishes (photographed hourly). The standard deviation of the computer to the mean human score was 1.32 h with the human scores lagging behind the automated germination curve. This demonstrates that an imperfect trueness of a computer vision system is not necessarily a problem, when the time to germinate is taken into account. Therefore, because software that considers image time would still not have scored individual images in complete agreement with a human scorer, the *k*-NN method described, which has high but imperfect trueness to the human score, is effective at scoring seeds on an image-by-image basis.

The technique investigated in this study could be used for high throughput imaging, particularly where the identification of individual germinated seed is of importance. This simple machine learning method could be refined by further optimisation of the *k*-NN, or substitution and optimisation using support vector machines (SVM) or random forest at the data categorisation stage. To go further, convolutional neural networks [37] have become the cutting edge of image categorisation in recent years but further work would be needed to optimise this more complex methods. The image dataset used in this study has been used with a convolutional neural network [38], and produced a similar accuracy when compared with the *k*-NN method but with higher computational demands; this could with refinement provide another direction for further study.

The *k*-NN method could also be expanded; for example [39] used the analysed properties of the seed/seedling image after germination to measure early seedling elongation. Commercially, seedling rates are calculated to produce an anticipated number of plants per unit area of seed sown. It is likely the approach developed will be utilised to rapidly screen the germination potential of new seed batches before widespread deployment to determine if oversowing is required to maintain crop densities.

## Abbreviations

ROC: receiver operating characteristic
AUC: area under curve
*k*-NN: *k* nearest neighbour
SVM: support vector machine
RGB: red, green and blue
HSB: hue, saturation, brightness
